# Protein Quantity and Quality of Safflower Seed Improved by NP Fertilizer and Rhizobacteria (*Azospirillum* and *Azotobacter* spp.)

**DOI:** 10.3389/fpls.2016.00104

**Published:** 2016-02-22

**Authors:** Asia Nosheen, Asghari Bano, Humaira Yasmin, Rumana Keyani, Rabia Habib, Syed T. A. Shah, Rabia Naz

**Affiliations:** ^1^Department of Biosciences, COMSATS Institute of Information TechnologyIslamabad, Pakistan; ^2^Department of Plant Sciences, Quaid-i-Azam UniversityIslamabad, Pakistan

**Keywords:** safflower, seed protein, amino acid, fertilization, phytohormones, SDS-PAGE

## Abstract

**HIGHLIGHTS**

- Rhizobacteria (*Azotobacter* spp.) have improved the quality and quantity of safflower seed protein.
- Protein quality was confirmed by SDS-PAGE and new bands were found in response to different combinations of rhizobacteria and lower doses of fertilizers.
- The PGPR application has reduced the use of fertilizers upto 50%.

Protein is an essential part of the human diet. The aim of this present study was to improve the protein quality of safflower seed by the application of plant growth promoting rhizobacteria (PGPR) in combination with conventional nitrogen and phosphate (NP) fertilizers. The seeds of two safflower cultivars Thori and Saif-32, were inoculated with *Azospirillum* and *Azotobacter* and grown under field conditions. Protein content and quality was assessed by crude protein, amino acid analysis, and SDS-PAGE. Seed crude protein and amino acids (methionine, phenylalanine, and glutamic acid) showed significant improvements (55–1250%) by *Azotobacter* supplemented with a quarter dose of fertilizers (BTQ) at *P* ≤ 0.05. Additional protein bands were induced in Thori and Saif-32 by BTQ and BTH (*Azotobacter* supplemented with a half dose of fertilizer) respectively. The *Azospirillum* in combination with half dose of fertilizer (SPH) and BTQ enhanced both indole acetic acid (IAA) (90%) and gibberellic acid (GA) (23–27%) content in safflower leaf. Taken together, these data suggest that *Azospirillum* and *Azotobacter* along with significantly reduced (up to 75%) use of NP fertilizers could improve the quality and quantity of safflower seed protein.

## Introduction

Oil seed crops are important sources of nutrients and can serve as a significant dietary source to meet nutritional requirements (Escudero et al., 2006; Yeilaghi et al., 2012). Consumption of oil seeds and legumes alone may meet the demand for protein and fat. Such practices have great potential for ensuring adequate nutrient and energy intake by infants and children in poor settings where protein-energy malnutrition (PEM) has continued to hamper optimal growth and development. With increasing global demand for livestock products, research into locally available food with a potential use as additional sources of protein and energy is imperative.

Seed proteins play a significant role in human nutrition and animal food. In plants, amino acids are the major building block for the synthesis of protein (Lam et al., 1996) and thus play an important role in human nutrition. Phenylalanine is an essential amino acid as it is a precursor of a very important metabolic compounds, namely phenylpropanoids. Similarly, tryptophan is of utmost importance as a precursor of phytohormones such as indole acetic acid **(**IAA). Therefore, it is critical that we improve seed protein quality through sustainable measures, such as through the application of plant growth promoting rhizobacteria (PGPR).

The PGPR of the genus *Azospirillum* and *Azotobacter* are widely distributed in the rhizosphere of tropical and subtropical plants. The mechanisms by which *Azospirillum* spp. can exert a positive influence on plant growth is probably via multiple responses including changes in the synthesis of phytohormones and nitrogen fixation, as well as nitrate reductase activity (El-Komy et al., 2003). Several mechanisms have been proposed by which they promote plant growth. These mechanisms include phytohormone production, stimulation of nutrient uptake, nitrogen fixation, improving the availability of primary nutrients to the plant (Wu et al., 2005), production of enzymes, riboflavin, thiamin, and the synthesis of antibiotics and fungicidal compounds (Bharathi et al., 2004). Mirzaei et al. (2010) reported that *Azospirillum* and *Azotobacter* inoculation improved the seed protein content of safflower compared to controls. Babalola (2010) reported that amino acid synthesis is an important feature of PGPR and amino acids synthesized by PGPR includes glutamic acid, lysine, valine, serine, isoleucine, and leucine.

Safflower is broadleaf oilseed crop of the family Asteraceae, predominantly adapted to dry land (Bahrami et al., 2014). It originated in southern Asia and is cultivated in China, India, Persia, Egypt, and Pakistan. It is used as a source of dye, medicines and food. It is cultivated as a source of oil and protein. It contains 34% oil and 22–24% protein and its seeds are rich source of natural antioxidant (tocopherol). The seed meal of safflower seeds after oil extraction is utilizd as a cattle feed and organic fertilizer.

With the increasing problem of soil/environmental pollution and leaching of nutrients, urgent action is needed to tackle the global threat of nitrogen pollution. Agronomic, biochemical, and biomass information about safflower yield in response to PGPR and NP fertilizers have been previously published (Nosheen and Bano, 2014). The main objective of this study was to minimize the use of nitrogen and phosphorus fertilizers by supplementing PGPR (*Azospirillum* and *Azotobacter*) to improve the protein quality of safflower seed, which is considered an important crop from a nutritional perspective.

## Materials and methods

A field experiment was conducted under natural conditions during October 2009–2010 and 2010–2011 in the field of the Department of Plant Sciences, Quaid-i-Azam University. A randomized complete block design (RCBD) with three replications was used with a plot size of 1 × 1 m^2^. The distance between the rows was 45 cm. Certified seeds of safflower cv. Thori and cv. Saif-32 was obtained from the National Agricultural Research Centre (NARC) Islamabad. Seeds were surface sterilized prior to sowing with 95% ethanol then sterilized with 10% chlorox for 3 min and washed successively 3–4 times with autoclaved distilled water.

### Seed inoculation

Liquid cultures of *Azospirillum brasilense* and *Azotobacter vinelandii* Khsr1 were grown at 24°C in Luria-Bertani (LB) medium. The PGPR were applied as seed inoculation at the rate of 10^6^ cells/ml. For inoculum preparation, 100 mL of LB media was inoculated with 24 h liquid cultures of *A. brasilense* (Accession no. GQ255949) and *A. vinelandii* Khsr1 (Accession no. GQ849485) and kept shaking (Excella E24, New Brunswick Scientific Incubator shaker Series, New Gersey, USA) for 72 h at 124 rpm at 24°C. The liquid cultures were centrifuged at 2415 *g* for 10 min. Supernatant was discarded and the pellet was diluted with autoclaved distilled water to an optical density at 600 nm. Sterilized seeds were soaked in liquid cultures for 6 h prior to sowing.

### Application of fertilizers

The fertilizers used were nitrogen and phosphorus (NP), urea was used as a source of nitrogen fertilizer and DAP (Diammonium phosphate) was used as a source of phosphorus fertilizer. Nitrogen fertilizers (N) were applied in three doses i.e., full dose of Urea (Urea 60 Kg ha^−1^), half (Urea 30 Kg ha^−1^), and quarter doses (Urea 15 Kg ha^−1^). The entire amount of phosphorus fertilizer (P) (full 30 kg ha^−1^, half 15 Kg ha^−1^ and quarter dose 7.5 Kg ha^−1^) was applied at the time of sowing while urea was applied at three different stages at an interval of 40 d, the first dose was applied at the time of sowing.

Due to its deep root zone, safflower crops can get moisture from well below the surface. During the season, 2–3 rounds of irrigation were applied. The first irrigation round was provided 1–1/2–2 months after germination; the second irrigation occurred at flowering time and last round of irrigation was given during seed development. A surface irrigation system was used until the field was saturated.

Following treatments were applied.

**Table:** 

**Treatments**	**Symbols**
Control (Without inoculation and without NP fertilizers)	C
NP fertilizers full recommended dose (Urea 60 Kg ha^−1^ and DAP 30 Kg ha^−1^)	CFF
NP fertilizers half dose (Urea 30 Kg ha^−1^ and DAP 15 Kg ha^−1^)	CFH
NP fertilizers quarter dose (Urea 15 Kg ha^−1^ and DAP 7.5 Kg ha^−1^)	CFQ
Single inoculation of *Azospirillum brasilense*	SP
*A. brasilense*+full dose of NP fertilizers	SPF
*A. brasilense*+half dose of NP fertilizers	SPH
*A. brasilense*+quarter dose of NP fertilizers	SPQ
Single inoculation of *Azotobacter vinelandii*	BT
*A. vinelandii*+full dose of NP fertilizers	BTF
*A. vinelandii*+half dose of NP fertilizers	BTH
*A. vinelandii*+quarter dose of NP fertilizers	BTQ

### Extraction and purification of phytohormones (IAA and GA)

Extraction and purification of phytohormones was done according to the method of Kettner and Doerffling (1995). Fresh leaves (1 g) were collected at the vegetative stage and ground in 80% methanol at 4°C with butylated hydroxyl toluene (BHT), used as an antioxidant. The extraction was done at 4°C till 72 h in dark with subsequent change of solvent at each 24 h. The extracted samples were centrifuged and the supernatant was reduced to aqueous phase using rotary thin film evaporator (RFE) at 35°C. The pH of the aqueous phase was adjusted to 2.5–3.0 with 0.1 N HCl and partitioned four times with 1/2 volume of ethyl acetate. The ethyl acetate was dried down completely using rotary thin film evaporator. The dried samples were re-dissolved in 1 mL of methanol (100%) and were analyzed on HPLC (Agilent 1100, Germany) using U.V. detector and C-18 column (39 × 300 mm).

For the identification of hormones, samples were filtered through millipore filters (0.45 μm) and injected onto the column. Methanol, acetic acid, and water (30:1:70) were used as a mobile phase. Wavelengths used for the detection of IAA was 280 nm (Sarwar et al., 1992), whereas for GA analysis it was adjusted to 254 nm (Li et al., 1994). These growth hormones were identified on the basis of retention time and peak area of the standards. Pure IAA and GA3 (Sigma Chemicals Co. Ltd. USA) were used as standards for the identification and quantification of plant hormones.

### Estimation of seed crude protein

Crude protein from seeds was estimated according to the method of Kjeldahl (AOAC, 2000). Seed samples (700 mg) were placed into Kjeldahl Digestion tubes and 5 g of each of K_2_SO_4_ and CuSO_4_ were added, then 25 mL of H_2_SO_4_ was added to the mixture. The mixture was digested for 1 h at 340°C. After cooling at room temperature, 20 mL of deionized water was added and after addition of 40% NaOH (25 mL) distillation was carried out. The liberated ammonia was collected in boric acid and titrated with HCl (0.1 N). A prepared blank was also treated using the same procedure. The crude protein percentage was calculated according to the following formula.

$$\begin{array}{l} \text{Crude protein}\left(\text{\%}\right)=\left(\text{sample titer}-\text{blank titer}\right)\times 14\times 5.30\\ \times 100∕\text{sample weight} \end{array}$$

Where, 14 is molecular weight of nitrogen and 5.30 is the nitrogen factor for safflower seed protein (Mosse, 1990).

### Amino acid analyses of seed

Quantitative analysis of amino acids was carried out according to the method of Tkachuk and Irvine (1969). Seed samples (20 mg) were placed in Pyrex test tubes and 4 mL of double distilled hydrochloric acid (6 N) was added and the mixture was frozen at −80°C. The hydrolysis of samples was done at 110°C for different time periods viz. 24, 48, and 72 h in an oven. Thereafter, the hydrochloric acid was removed using a dessicator containing sodium hydroxide pellets. Subsequently 25 mL of citrate buffer of normality 0.2 and pH 2.2 containing octanoic acid and Brij-35 were added and insoluble humin was removed by vacuum filtration. The supernatant (filtrate) was used for amino acid analysis using an amino acid analyzer. A Hitachi L-8900 Automatic Amino Acid Analyzer (Hitachi High-Technologies Corporation, Tokyo, Japan) with a 4.6 (ID) × 60 mm ion exchange column was used to determine the amino acid profiles of the samples. The following analyzer settings were used for the analysis: buffer flow rate of 0.4 mL/min, reagent flow rate of 0.35 mL/min, reactor heater temperature of 135°C, column temperature of 75°C, auto-sampler temperature of 5 ~ 8°C, run time of 35.3 (sulfur-containing amino acids) or 56.3 min (all other amino acids), sample injection volume of 20 μL, and detection wavelength of 570 (proline) or 440 nm (all other amino acids). Protein hydrolysate buffer set (Kanto Chemical Co., Inc., Tokyo, Japan) and hydrochloric acid was used as the mobile phase solvents. A standard amino acid mixture of cysteic acid and methionine sulfone (20 μL/mL) was diluted to 100 μmol/L for amino acid quantification and calibration. Amino acid concentrations are reported in g/100 g.

### Statistical analysis

Data were analyzed by Statistix software version 8.1 using factorial design of analysis of variance (ANOVA) (Table 1). Mean values were compared according to Steel and Torrie (1980) by least significant difference (LSD) at *P* < 0.05. Linear regression analysis was conducted to determine the relationship between seed protein and phytohormones (IAA, GA) and amino acid. The correlation among different factors was assessed by the Linear Regression/Pearson Correlation Coefficient test using OriginPro 2016 (OriginLab, Northampton, MA).

**Table 1:** **Analysis of variance for various parameters of safflower used in this study**.

**Variables**	**Source**	**DF**	**SS**	**MS**	***F***	***P*-Value**
Gibberellic acid (Year 1)	Replicates	2	1.79	0.893		
	Treatments	11	7258.76	659.887	3340.28	0.000
	Varieties	1	25.20	25.205	127.59	0.000
	Treatments ^*^ Varieties	11	208.77	18.979	96.07	0.000
Gibberellic acid (Year 2)	Replicates	2	1.58	0.790		
	Treatments	11	6732.89	612.081	3550.06	0.000
	Varieties	1	7.62	7.625	44.22	0.000
	Treatments ^*^ Varieties	11	1379.67	125.425	727.46	0.000
Indole acetic acid (Year 1)	Replicates	2	2.2	1.09		
	Treatments	11	18593.7	1690.34	1083.92	0.000
	Varieties	1	3185.5	3185.48	2042.68	0.000
	Treatments ^*^ Varieties	11	1905.0	173.18	111.05	0.000
Indole acetic acid (Year 2)	Replicates	2	1.9	0.96		
	Treatments	11	30317.8	2756.16	5253.16	0.000
	Varieties	1	17.2	17.21	32.80	0.000
	Treatments ^*^ Varieties	11	408.4	37.13	70.77	0.000
Seed Protein (Year 1)	Replicates	2	0.877	0.439		
	Treatments	11	419.052	38.096	206.56	0.000
	Varieties	1	295.448	295.448	1601.98	0.000
	Treatments ^*^ Varieties	11	133.616	12.147	65.86	0.000
Seed Protein (Year 2)	Replicates	2				
	Treatments	11	99.631	9.057	17672.9	0.000
	Varieties	1	509.284	509.284	993724	0.000
	Treatments ^*^ Varieties	11	97.540	8.867	17302.0	0.000
Methionine	Replicates	2				
	Treatments	11	0.73296	0.06663	97.69	0.000
	Varieties	1	0.03092	0.03092	45.33	0.000
	Treatments ^*^ Varieties	11	1.09576	0.09961	146.05	0.000
Tyrosine	Replicates	2				
	Treatments	11	0.86582	0.07871	247.07	0.000
	Varieties	1	0.10765	0.10765	337.90	0.000
	Treatments ^*^ Varieties	11	0.75641	0.06876	215.84	0.000
Phenylalanine	Replicates	2				
	Treatments	11	0.71494	0.06499	73.21	0.000
	Varieties	1	0.16236	0.16236	182.88	0.000
	Treatments ^*^ Varieties	11	0.76154	0.06923	77.98	0.000
Proline	Replicates	2				
	Treatments	11	0.08215	0.00747	15.47	0.000
	Varieties	1	1.901E-07	1.901E-07	0.00	0.984
	Treatments ^*^ Varieties	11	0.12216	0.01111	23.01	0.000
Lysine	Replicates	2				
	Treatments	11	0.16788	0.01526	42.88	0.000
	Varieties	1	0.18665	0.18665	524.37	0.000
	Treatments ^*^ Varieties	11	0.13614	0.01238	34.77	0.000
Histidine	Replicates	2				
	Treatments	11	0.60677	0.05516	102.43	0.000
	Varieties	1	0.23165	0.23165	430.15	0.000
	Treatments ^*^ Varieties	11	0.64360	0.05851	108.64	0.000
Glutamic acid	Replicates	2				
	Treatments	11	7.0857	0.64416	65.80	0.000
	Varieties	1	0.0004	0.00039	0.04	0.843
	Treatments ^*^ Varieties	11	9.1814	0.83468	85.27	0.000
Glycine	Replicates	2				
	Treatments	11	0.09513	0.00865	15.47	0.000
	Varieties	1	0.00316	0.00316	5.65	0.021
	Treatments ^*^ Varieties	11	0.07332	0.00667	11.92	0.000

### Protein profiling by SDS-PAGE

The protein profile in seeds was analyzed by SDS-PAGE (Sodium Dodecyl Sulfate-Polyacrylamide Gel Electrophoresis) according to Laemmli (1970) on a Biorad Protean II system.

Safflower seeds were ground to a fine powder using a pestle and mortar. A total of 0.01 g of the powdered sample was weighed in 1.5 mL eppendorf tube, 400 μL protein extraction buffer (0.05 M Tris-HCl pH 8.0, 0.2% SDS, 5 M Urea, 1% ß-mecaptoethanol) was added and vortexed for 2 min. For purification, the homogenate was centrifuged at 4°C for 10 min at 2415 *g* to collect supernatant and remove the residue. It was ensured that no cell debris was taken, a Hamilton syringe was used to carefully collect the supernatant and avoiding the oil layer. This ensured the sample was clean before loading on the gel.

Prior to the preparation of the gel, the glass plates were cleaned with 70% ethanol. Two gels were prepared, first a separating gel (12.25%) was prepared and poured between the two glass plates and after 30–40 min a stacking gel (4.5%) was poured. The gels were placed into an electrophoresis tank and electrode buffer (0.025 M Tris, 0.129 M Glycine, 0.125% SDS) was added. A protein marker (Fermentas, protein ladder) 5 μL and sample (10 μL) were loaded. The voltage was 180 for 50–55 min at 100 mA.

Gels were removed after electrophoresis and transferred into a tray containing staining solution (Coomassie Brilliant Blue G-250, methanol, acetic acid, and distilled water) for 40 min on a shaker at 40 rpm, and then destained with destaining solution (Methanol, acetic acid, distilled water). Gel analysis was conducted using a gel documentation system (Bio-Rad, Italy).

## Results

### Effect of PGPR and NP fertilizers on leaf GA and IAA

The effect of *Azospirillum, Azotobacter*, and fertilizers on the phytohormone concentration was determined on a fresh weight basis. Results indicated that all treatments of PGPR and fertilizers significantly increased the gibberellic acid (GA) contents in both varieties during two years (Table 2). During 2009–2010, a maximum increase (27%) in leaf GA contents was recorded with *Azospirillum* in combination with a half dose of NP fertilizers (SPH) treatment in cv. Thori. In cv. Saif-32, the highest increase (23%) in GA content was recorded with *Azotobacter* in combination with a half dose of NP fertilizers (BTH) treatment. During the second year (2010–2011) similar patterns of increased GA content was observed.

**Table 2:** **Mean comparison of leaf gibberellic acid and indole acetic acid contents affected by PGPR and NP fertilizer treatments in safflower**.

**Treatments**	**Gibberellic acid (μg.g^−1^)**				**Indole acetic acid (μg.g^−1^)**			
	**2009–2010**		**2010–2011**		**2009–2010**		**2010–2011**	
	**Thori**	**Saif-32**	**Thori**	**Saif-32**	**Thori**	**Saif-32**	**Thori**	**Saif-32**
C	141.60 s	145.07 r	145.47 s	142.33 t	38.80 r	40.34 qr	60.53 u	65.33 t
CFF	155.50 p	153.53 q	160.40 j	149.23 r	45.43 o	44.40 op	75.93 s	76.00 s
CFH	158.00 n	156.53 o	155.52 mn	156.23 m	42.37 pq	63.37 jk	88.33 o	91.63 n
CFQ	156.80 o	156.47 o	150.93 pq	151.07 p	40.73 qr	57.37 m	85.53 p	95.27 m
SP	160.53 m	161.50 l	163.47 i	155.33 n	55.83 m	70.13 i	80.70 r	82.37 q
SPF	163.27 k	165.50 j	167.33 g	159.37 k	53.70 n	73.40 h	101.57 k	97.50 l
SPH	181.03 a	177.50 d	181.03 a	179.37 b	80.60 f	100.67 a	128.37 b	130.33 a
SPQ	169.47 h	172.63 f	163.67 i	173.20 d	65.37 j	88.03 d	120.73 e	115.60 g
BT	158.53 n	163.30 k	150.37 pq	150.33 q	71.33 i	60.63 l	106.47 j	112.70 h
BTF	165.50 j	166.27 i	153.33 o	165.97 h	75.10 h	90.53 c	123.20 d	118.50 f
BTH	170.50 g	179.37 b	157.57 l	177.50 c	77.63 g	95.47 b	125.80 c	129.33 ab
BTQ	178.37 c	175.63 e	171.50 e	168.40 f	62.33 kl	84.53 e	113.43 h	107.77 i
LSD value	0.7305		0.7308		2.0372		1.2093	

*Means with at least one common letter are not significantly different at the P = 0.05. Detail of treatments as described below*.

*C, Control; CFF, NP fertilizers full dose; CFH, NP fertilizers half dose; CFQ, NP fertilizers quarter dose; SP, A. brasilense; SPF, A. brasilense+full dose of NP fertilizers; SPH, A. brasilense+half dose of NP fertilizers; SPQ, A. brasilense+quarter dose of NP fertilizers; BT, A. vinelandii; BTF, A. vinelandii+full dose of NP fertilizers; BTH, A. vinelandii+half dose of NP fertilizers; BTQ, A. vinelandii+quarter dose of NP fertilizers*.

During 2009–2010 and 2010–2011, the application of PGPR and NP fertilizers significantly improved the IAA content of safflower leaves as compared to the untreated control (Table 2). During both years, the maximum significant increase (107%) was found in the SPH treatment. In cv. Saif-32, the increase in IAA content was similar to that of cv. Thori.

### Effect of PGPR and NP fertilizers on seed crude protein and methionine contents

During the first year, the maximum increase (62%) in seed crude protein was recorded in the BTQ treatment when compared with the control in cv. Thori. This increase was 23% and 13% higher in BTQ as compared to BT and CFQ treatments respectively (Table 3). In the case of cv. Saif-32, a maximum increase (6%) in seed crude protein was recorded in BTQ over the control. The data for the second year showed similar patterns with some variations among the treatments but with a maximum increase observed in BTQ treatment as seen in first year.

**Table 3:** **Mean comparison of seed crude protein and methionine affected by PGPR and NP fertilizer treatments in safflower**.

	**Seed crude protein (%)**				**Methionine (g.100g^−1^)**	
**Treatments**	**2009–2010**		**2010–2011**	
	**Thori**	**Saif-32**	**Thori**	**Saif-32**	**Thori**	**Saif-32**
C	13.14 k	22.46 c	14.84 u	22.96 f	0.36 fg	0.27 kl
CFF	18.81 fg	23.27 b	14.46 v	23.01 e	0.54 b	0.32 ghij
CFH	16.33 i	18.53 g	17.06 q	21.97 k	0.49 c	0.30 ijkl
CFQ	18.53 g	17.66 h	19.26 o	22.08 j	0.35 gh	0.58 b
SP	16.10 i	17.40 h	17.47 p	23.22 d	0.36 fg	0.29 jkl
SPF	15.33 j	23.42 ab	19.24 o	22.54 i	0.45 d	0.27 kl
SPH	11.23 l	16.40 i	15.35 t	21.09 n	0.47 cd	0.26 l
SPQ	17.56 h	20.30 e	15.81 s	23.98 a	0.14 m	0.40 ef
BT	16.33 i	20.66 de	16.36 r	23.60 b	0.43 de	0.31 hijk
BTF	20.63 de	23.82 ab	21.16 m	22.79 g	0.56 b	0.28 kl
BTH	13.36 k	19.40 f	17.08 q	22.59 h	0.53 b	0.34 ghi
BTQ	21.33 d	24.00 a	21.24 l	23.33 c	0.40 ef	0.97 a
LSD value	0.7058		0.0372		0.0429	

*Means with at least one common letter are not significantly different at the P = 0.05. Detail of treatments as described below*.

*C, Control; CFF, NP fertilizers full dose; CFH, NP fertilizers half dose; CFQ, NP fertilizers quarter dose; SP, A. brasilense; SPF, A. brasilense+full dose of NP fertilizers; SPH, A. brasilense+half dose of NP fertilizers; SPQ, A. brasilense+quarter dose of NP fertilizers; BT, A. vinelandii; BTF, A. vinelandii+full dose of NP fertilizers; BTH, A. vinelandii+half dose of NP fertilizers; BTQ, A. vinelandii+quarter dose of NP fertilizers*.

In cv. Thori, the highest increase (55%) in methionine content was recorded in the BTF treatment, the value of which was 18% higher over BT treatment (Table 3). In cv. Saif-32, maximum increase (259%) was recorded in BTQ treatment.

### Effect of PGPR and NP fertilizers on phenylalanine, glutamic acid and glycine contents

The maximum percentage increase in phenylalanine content (1250%) was recorded in the BTQ treatment over the control which was statistically similar to the BTH treatment in cv. Thori (Table 4). Treatment BTQ showed a 61 and 77% increase over BT and CFQ treatments respectively. In the case of cv. Saif-32, treatments SP and BTH exhibited significant increases in phenylalanine content.

**Table 4:** **Mean comparison of phenylalanine, glutamic acid, glycine contents affected by PGPR and NP fertilizer treatments in safflower**.

**Treatments**	**Phenylalanine (g.100g^−1^)**		**Glutamic acid (g.100g^−1^)**		**Glycine (g.100g^−1^)**	
	**Thori**	**Saif-32**	**Thori**	**Saif-32**	**Thori**	**Saif-32**
C	0.04 lm	0.18 g	0.73 e	0.45 fg	0.04 j	0.04 j
CFF	0.48 b	0.13 hi	0.79 e	0.44 fg	0.14 cde	0.21 a
CFH	0.05 klm	0.18 g	0.77 e	0.51 f	0.17 bc	0.10 fgh
CFQ	0.12 ij	0.08 jkl	0.71 e	0.26 h	0.11 efg	0.05 ij
SP	0.03 m	0.33 d	0.46 fg	1.36 c	0.13 def	0.10 fgh
SPF	0.42 c	0.17 gh	1.08 d	0.76 e	0.17 abc	0.12 def
SPH	0.08 jkl	0.13 hi	0.23 h	0.16 hi	0.08 ghi	0.12 ef
SPQ	0.26 e	0.17 gh	1.54 b	1.16 d	0.18 ab	0.11 efg
BT	0.21 fg	0.09 ijk	1.65 b	0.69 e	0.18 ab	0.10 efgh
BTF	0.40 c	0.12 ij	1.07 d	0.68 e	0.18 ab	0.09 fgh
BTH	0.51 ab	0.23 ef	0.04 i	1.07 d	0.04 j	0.12 ef
BTQ	0.54 a	0.19 fg	0.31 gh	1.88 a	0.07 hij	0.16 bcd
LSD value	0.0489		0.1624		0.0388	

*Means with at least one common letter are not significantly different at the P = 0.05. Detail of treatments as described below*.

*C, Control; CFF, NP fertilizers full dose; CFH, NP fertilizers half dose; CFQ, NP fertilizers quarter dose; SP, A. brasilense; SPF, A. brasilense+full dose of NP fertilizers; SPH, A. brasilense+half dose of NP fertilizers; SPQ, A. brasilense+quarter dose of NP fertilizers; BT, A. vinelandii; BTF, A. vinelandii+full dose of NP fertilizers; BTH, A. vinelandii+half dose of NP fertilizers; BTQ, A. vinelandii+quarter dose of NP fertilizers*.

The glutamic acid contents were significantly increased in SPF, SPQ, BT, and BTF treatments as compared to that of the control in cv. Thori (Table 4). Treatment BT showed a maximum increase (126%) in glutamic acid content over the control. In cv. Saif-32, a maximum increase was observed in the BTQ treatment which showed 63 and 86% significant increases over BT and CFQ treatments respectively.

All the treatments had significant effects on the glycine content except BTH and BTQ treatments which showed a non-significant effect as compared to that of the control in cv. Thori (Table 4). The treatments SPQ, BT, and BTF were statistically similar and showed a maximum increase (350%) as compared to the control. In cv. Saif-32, the CFF treatment exhibited a higher increase (425%) over the control.

### Proline, tyrosine, histidine, and lysine contents

A maximum percentage increase (136%) in proline content was observed in the CFF treatment over the control in cv. Thori (Table 5). Treatment SP showed a 9% increase whereas BT showed a 54% reduction as compared to that of the control. In cv. Saif-32, treatments SP, SPF, SPH, and BTF showed significant increases whereas, the rest of the treatments showed non-significant differences in proline content as compared to the control.

**Table 5:** **Mean comparison of proline, tyrosine, histidine, and lysine contents affected by PGPR and NP fertilizer treatments in safflower**.

**Treatments**	**Proline (g.100g^−1^)**		**Tyrosine (g.100g^−1^)**		**Histidine (g.100g^−1^)**		**Lysine (g.100g^−1^)**	
	**Thori**	**Saif-32**	**Thori**	**Saif-32**	**Thori**	**Saif-32**	**Thori**	**Saif-32**
C	0.11 ijk	0.14 ghi	0.11 kl	0.09 l	0.06 hij	0.04 jk	0.04 kl	0.07 hijk
CFF	0.26 a	0.16 efg	0.56 ab	0.17 j	0.12 ef	0.03 jk	0.26 b	0.05 jkl
CFH	0.16 efg	0.10 jkl	0.55 bc	0.27 h	0.09 fgh	0.03 jk	0.06 ijk	0.12 ef
CFQ	0.18 def	0.13 ghij	0.22 i	0.11 kl	0.08 ghi	0.22 d	0.08 ghij	0.04 kl
SP	0.12 hijk	0.19 cde	0.26 h	0.11 kl	0.06 hij	0.43 b	0.14 e	0.05 jkl
SPF	0.14 fgh	0.19 cde	0.53 c	0.17 j	0.15 e	0.05 hijk	0.29 a	0.09 fgh
SPH	0.09 kl	0.24 ab	0.31 g	0.48 d	0.02 k	0.23 d	0.22 cd	0.11 efg
SPQ	0.22 bc	0.11 ijk	0.31 g	0.59 a	0.11 fg	0.59 a	0.19 d	0.02 l
BT	0.05 m	0.14 ghi	0.36 e	0.25 h	0.15 e	0.33 c	0.14 e	0.07 ijk
BTF	0.18 def	0.19 cde	0.34 ef	0.16 j	0.09 fgh	0.03 jk	0.30 a	0.08 ghi
BTH	0.21 bcd	0.09 jkl	0.13 k	0.19 j	0.04 jk	0.05 ijk	0.22 c	0.09 fgh
BTQ	0.07 lm	0.11 ijk	0.17 j	0.33 fg	0.03 jk	0.33 c	0.11 efg	0.04 kl
LSD value	0.0361		0.0293		0.0381		0.0310	

*Means with at least one common letter are not significantly different at the P = 0.05. Detail of treatments as described below*.

*C, Control; CFF, NP fertilizers full dose; CFH, NP fertilizers half dose; CFQ, NP fertilizers quarter dose; SP, A. brasilense; SPF, A. brasilense+full dose of NP fertilizers; SPH, A. brasilense+half dose of NP fertilizers; SPQ, A. brasilense+quarter dose of NP fertilizers; BT, A. vinelandii; BTF, A. vinelandii+full dose of NP fertilizers; BTH, A. vinelandii+half dose of NP fertilizers; BTQ, A. vinelandii+quarter dose of NP fertilizers*.

All treatments significantly increased the tyrosine content in both varieties except BTH treatment in cv. Thori and CFQ and SP treatments in cv. Saif-32 (Table 5). The CFF treatment was highly responsive (409%) in increasing tyrosine content as compared to the control. In the case of cv. Saif-32, a maximum increase was recorded in the SPQ treatment over the control, the value was 81% higher over SP and CFQ treatments.

Results indicated that the treatments CFF, SPF, SPQ, and BT significantly improved the histidine content of seed as compared to that of untreated control in cv. Thori (Table 5). Treatment SPF and BT were statistically similar and exhibited a maximum increase (150%) over the control. In cv. Saif-32, a maximum percentage increase was observed in the SPQ treatment.

All the treatments significantly improved the lysine content of the seed except the CFH treatment which showed non-significant increase over the control in cv. Thori (Table 5). Treatment with BTF showed the highest increase (650%) as compared to that of the control with a 13 and 53% significant increase over CFF and BT treatments respectively. In cv. Saif-32, treatments CFH, SPF, and SPH showed significant increases as compared to the control. A maximum increase (71%) was recorded in CFH treatment over the control.

### Protein profile of safflower seed

**Figure 1:**
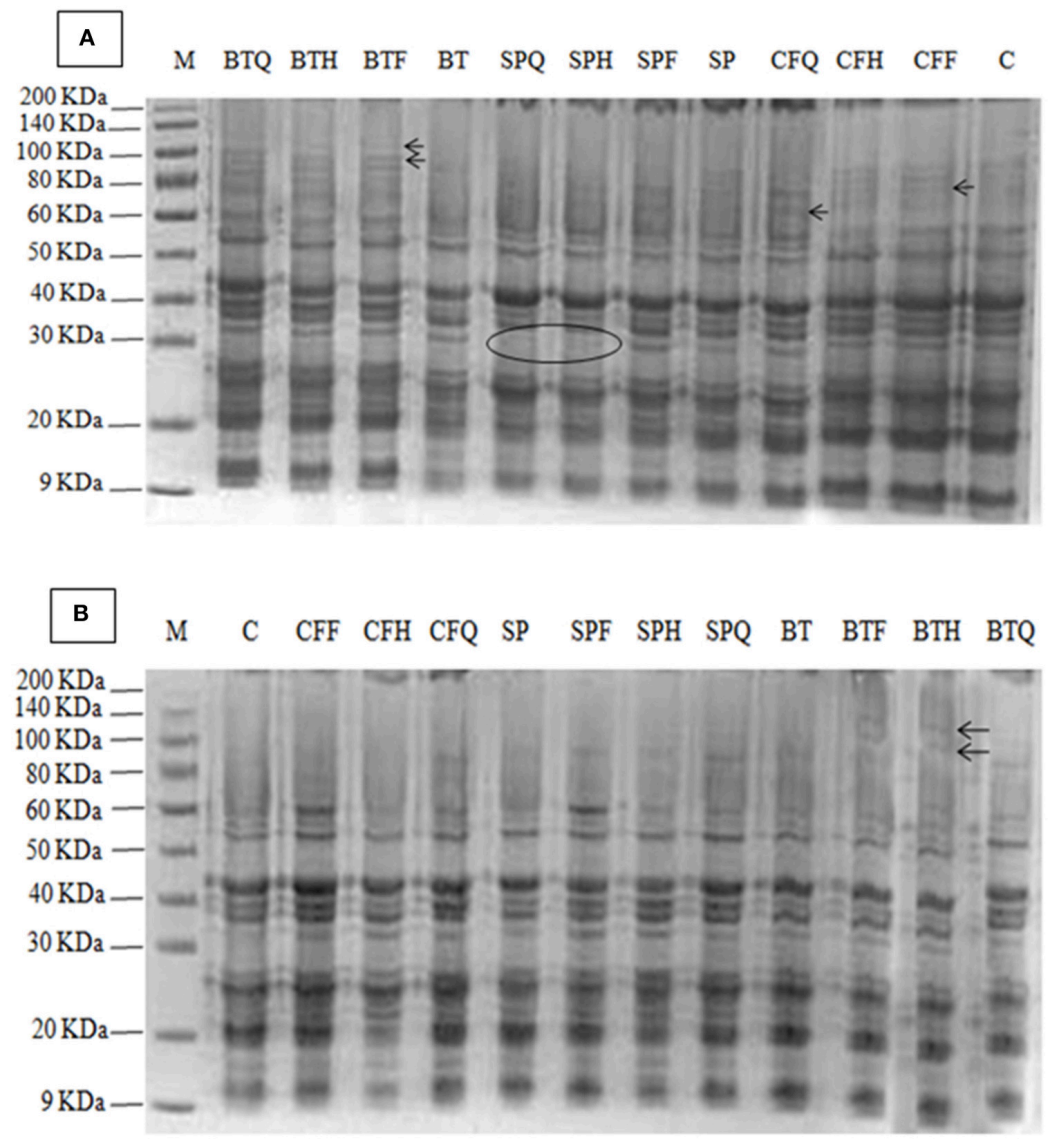
**(A)** Effect of PGPR and NP fertilizers on the protein profile of safflower cv. Thori, **(B)** Effect of PGPR and fertilizers on the protein profile of safflower in cv. Saif-32. The arrows indicated newly induced band and the circle indicated the absence of a band at that region. Detail of treatments as in Table 1.

The electrophoretic pattern of seed protein from safflower in cv. Thori differed in various treatments. A total of 20 bands were recorded in the seed protein of cv. Thori (Figure 1A). The highest number of protein bands (20) was recorded in the BTQ treatment. Two new bands of 130 and 100 KDa molecular weight were induced in BTF, BTH, and BTQ treatments. The treatments SP, SPF, SPH, SPQ, and BT induced a new polypeptide band of 60 Kda molecular weight which was absent in the control and the rest of the treatments. A polypeptide band of 29 Kda was present in all the treatments but absent in the SPH and SPQ treatments.

In cv. Saif-32, a total of 17 protein bands was present (Figure 1B). The highest number (16) of polypeptide bands was recorded in the BTF and BTH treatments. Two new polypeptide bands of molecular weight of 120 and 95 Kda were induced by BTF and BTH treatments respectively. Similarly, a new band of 80 Kda was induced by CFF, CFQ, SPF, SPH, SPQ, BT, BTF, and BTQ treatments and was absent in the control. Another band of molecular weight of 60 Kda was induced by CFF, CFQ, BTH, and BTQ treatments and was absent in the control. A polypeptide band of molecular weight 50 Kda was present in all the treatments but absent in the control and BTH treatment. Another new band of 20 Kda was observed in all the treatments except the control.

## Discussion

**Figure 2:**
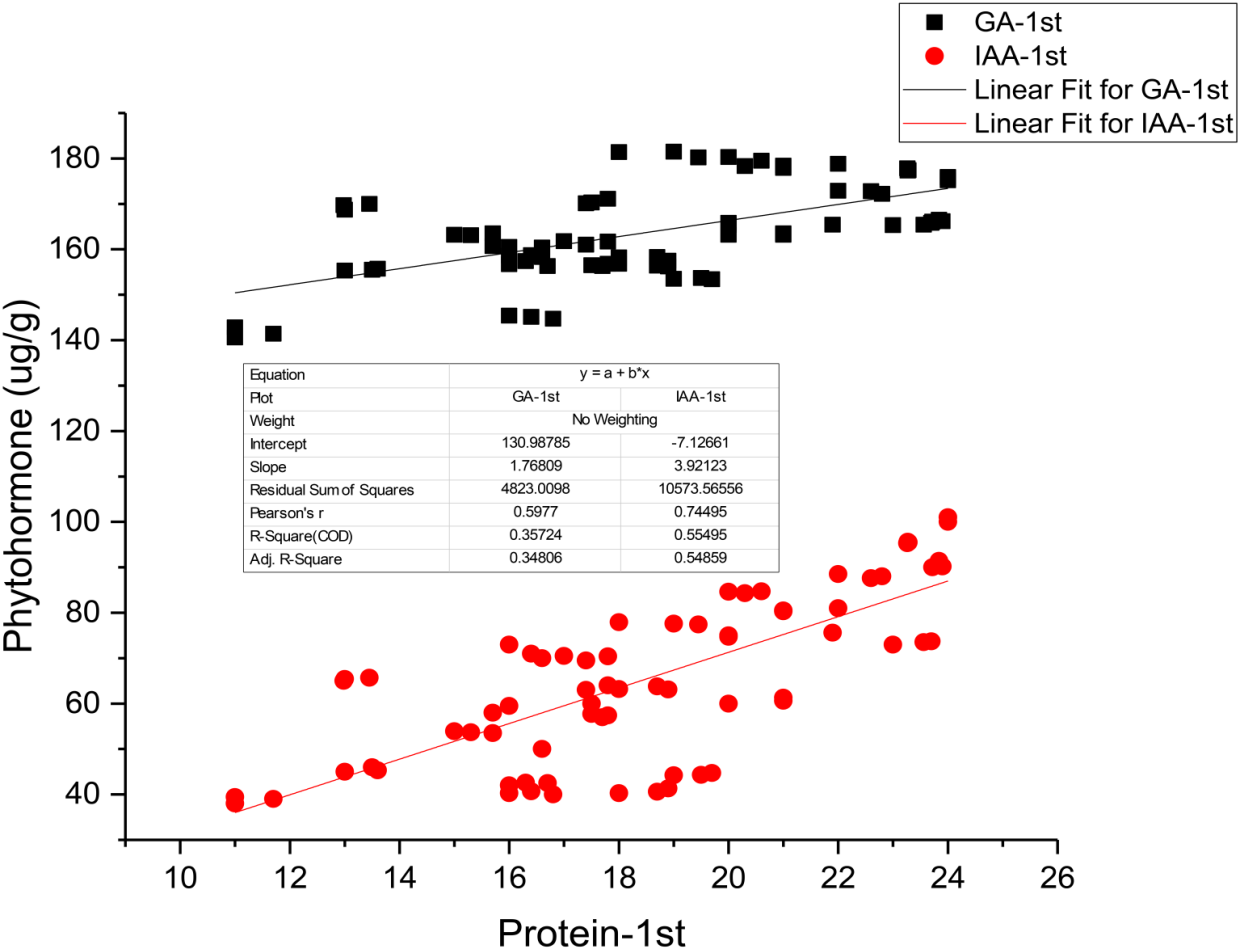
**Linear Regression analyses between seed protein and phytohormones (IAA and GA)**.

Applications of PGPR and NP fertilizers improve the growth, yield, and nutritive quality of safflower. In the present study, PGPR in combination with lower doses of NP fertilizers brought about significant increase in endogenous hormonal levels (IAA and GA) in safflower leaves. However, the highest increase was recorded with *Azospirillum* in combination with a half dose of NP fertilizers (SPH). This finding was in agreement with the findings of Glick (2012) who showed that PGPR increases the production of phytohormones such as IAA, GA, and cytokinin (Ck). Kiba et al. (2011) reported that phytohormones such as IAA, ABA, and Ck were closely linked to nitrogen signaling and provided insight that nitrogen and phytohormones signals were integrated in order to alter the morphology and physiology of plants. The present results are in agreement with those of Saharan and Nehra (2011) who reported that *Azospirillum, Azotobacter, Pseudomonas* increased the plant growth and yield by a variety of mechanisms, among those, one was the production of phytohormones. Lone et al. (2005) reported that phytohormones are the chief constituent of protein changes and can improve the yield and quality of oilseed crops. Our regression analysis also shows very strong relationship between seed protein and IAA and GA contents (Figure 2).

In the present study, a maximum increase in seed crude protein by BTQ treatment is in accordance with the finding of Mohsennia and Jalilian (2012) who reported that *Azotobacter chroccoccum* and *Azospirillum lipoferum* inoculation leads to the production of higher protein concentration in the achene of sunflowers. The PGPR increased seed protein, and as they are nitrogen fixers, the nitrogen can also be utilized for protein synthesis. Previous reports also showed that the application of PGPR influences the crude protein of safflower seed and improve the protein content (Stefan et al., 2013). In the present study, improvement in seed crude protein in full dose of fertilizers might be due to the increased supply of nitrates to the plant during photosynthesis and growth stages. This increase in nitrogen rate may increase the biosynthesis of amino acids and stimulate the accumulation of protein in the seed (Greef, 1994). These results are in concomitant with those of Hasanpour et al. (2012) who demonstrated that nitrogen fertilizers significantly enhance the protein level in sesame. Lone et al. (2005) reported that phytohormones are the chief constituent of protein levels and oil structure and improve the yield and quality of oilseed crops. In the present work, the PGPR in combination with different doses of fertilizers increased the endogenous phytohormone levels, with profound increments recorded in *Azospirillum* in the presence of half dose of fertilizers. This suggests in addition to nitrogen fertilizers, the production of phytohormones is also an important factor which enhances the seed crude protein.

**Figure 3:**
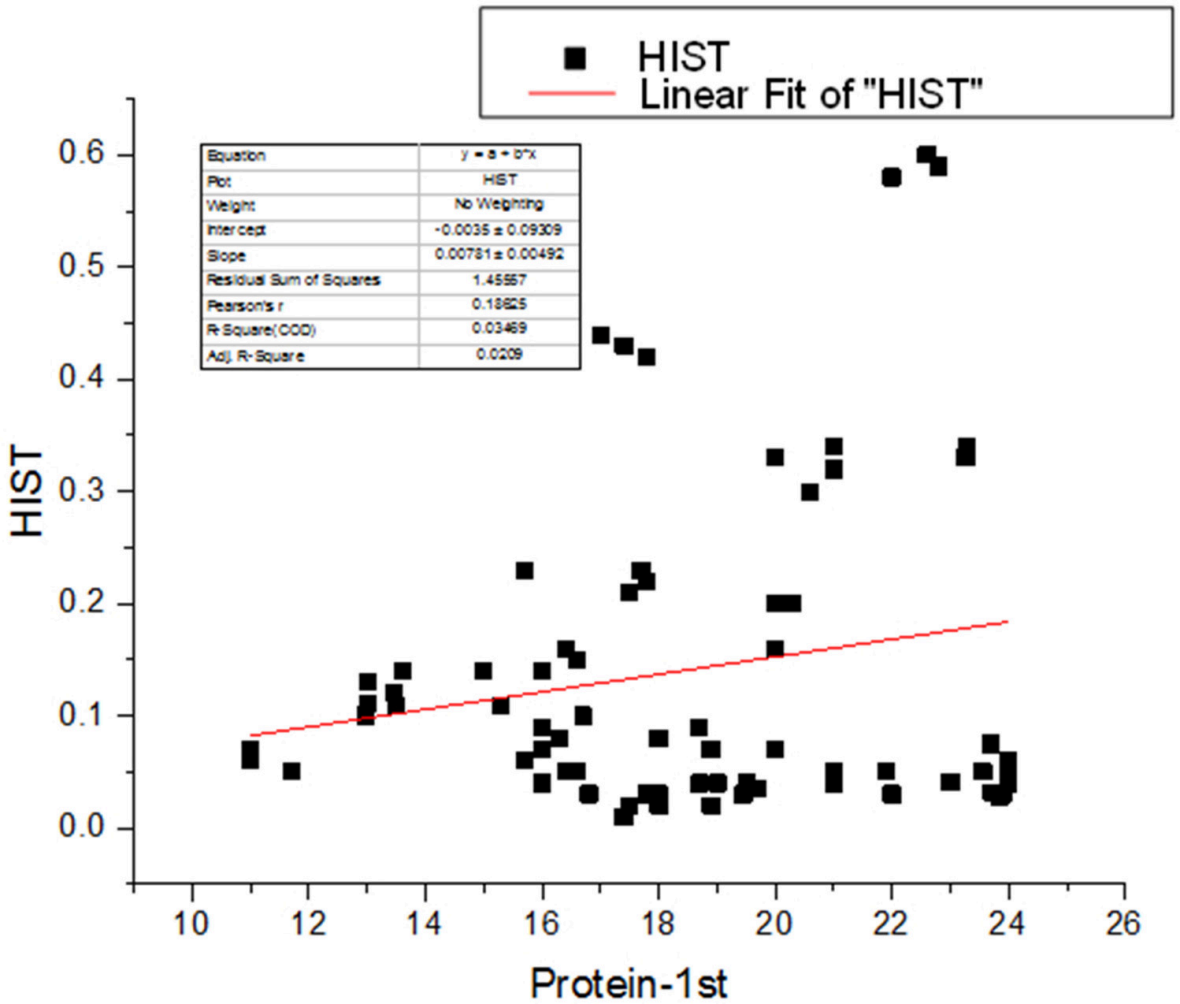
**Linear Regression analyses between seed protein and amino acid (histidine)**.

Amino acids are the building block for the synthesis of protein and their synthesis is an important feature of PGPR. The amino acids synthesized by PGPR include methionine, glutamine, glutamic acid, isoleucine, leucine, and aspartic acid (Babalola, 2010). During the present study methionine was significantly improved by the BTQ treatment, phenylalanine, glutamic acid, and glycine content was significantly augmented by BTH, SPQ, and CFF whereas, proline was increased by CFF treatment. A profound increase in tyrosine and histidine content was recorded by SPQ treatment. These results are in accordance to that of Kang et al. (2012) who reported that plants treated with the PGPR showed higher increases in crude protein and amino acids (threonine, alanine, and proline) in cucumber. Application of *A. calcoaceticus* brought about the increase in proline concentration which plays a pivotal role in osmotic adjustments (Evelin et al., 2009; Khan et al., 2011) and acts as a reserve of organic nitrogen that is available as a source of energy (Meloni et al., 2001). Similar results were reported by Hamdia et al. (2004) that inoculation of *Azospirillum* in maize increased amino acids such as methionine, proline glutamic, glycine, tyrosine, histidine, phenylalanine, and lysine. The NH_3_ synthesized by *Azospirillum* was incorporated into α-ketoglutarate to form glutamic acid (El-Komy et al., 2003); the elevated concentration of glutamic acid serves as a sink for the synthesis of protein and amino acids (Wang et al., 1999). It was reported by Kiba et al. (2011) that phytohormones regulate the acquisition of nitrogen and that the increase in nitrogen results in an increase in protein and amino acid synthesis. The linear regression analysis also shows a positive relationship between seed protein and amino acids (Figure 3). Akbari et al. (2011) also reported that the higher rate of nitrogen increased the amino acid synthesis in the leaves of *Helianthus annus* L. which stimulate protein accumulation in the seed.

There is not much information about the effects of PGPR on protein profiling of seed. In the present study we report that *Azotobacter*, as a single inoculant as well as in combination with different doses of fertilizers induces new bands in safflower seed; with the highest bands (130 and 100 KDa) being recorded in the BTQ treatment in cv. Thori., while 120 and 95 KDa bands in BTF and BTH respectively were observed in cv. Saif-32. The present results are in parallel to that of Shehata and El-Khawas (2003) who demonstrated that biofertilizer applications induce the protein bands of different molecular weights i.e., biogien induced a 2.1 KDa protein band and microbien induced 14.9 KDa protein band, which play a favorable role and may serve as an adaptive mechanism for the biofertilizers to improve the plant productivities and protein quality of the sunflower seed. The present results are in agreement with those of Prathibha and Siddalingeshwara (2013) and Selvakumar et al. (2012) who reported that PGPR (*Pseudomonas fluorescence, Bacillus subtilis, Rhizobium*, and *Phosphobacteria*) inoculation altered the protein profiling in seeds of sorghum and some new protein bands were found. The treatments of BTH and BTQ revealed 100% homology with each other in inducing protein bands and similar findings were reported by Shehata and El-Khawas (2003). The induction of new protein bands showed a strong correlation between the crude seed protein and new protein band as the similar treatments have parallel effect on both the traits.

The improvement in seed protein quality in terms of seed crude protein, amino acid composition and increase in number of protein bands by the application of *Azotobacter* in combination with quarter dose of NP fertilizers is an interesting finding. These treatments also improved the phytohormone content which are important structural building blocks of amino acids. We conclude that the use of *Azotobacter* significantly lowers the use of NP fertilizers while improving the quality and quantity of safflower seed protein which may have biological and economic impacts. However, further research is needed to confirm the interaction of phytohormones with protein and amino acids at molecular level. The classic biochemical, physiological, and genetic approaches may be combined with highly sensitive and high throughput phytohormone analysis and systems approaches to explore the molecular pathways associated with these interactions which in turn will take us closer to achieving our goal for better safflower seed protein.

## Author contributions

Dr. AB supervised the work. Dr. AN conducted research work and wrote manuscript. Dr. HY and Dr. RN helped in statistical analysis. Dr. RK and Dr. RH reviewed the manuscript. Dr. SS helped in statistical analysis and reviewed the manuscript.

### Conflict of interest statement

The authors declare that the research was conducted in the absence of any commercial or financial relationships that could be construed as a potential conflict of interest.
